# Peripheral blood cell signature and inflammatory responses during pregnancy-associated malaria

**DOI:** 10.1186/1475-2875-11-S1-P141

**Published:** 2012-11-09

**Authors:** Samad Ibitokou, Stéphanie Boström, Laurent Brutus, Nicaise T Ndam, Stefania Varani, Achille Massougbodji, Philippe Deloron, Marita Troye-Blomberg, Nadine Fievet, Adrian JF Luty

**Affiliations:** 1 Centre d’étude et de recherche sur le paludisme associé à la grossesse et à l’enfance (CERPAGE), Faculté des Sciences de la Santé, Université d’Abomey-Calavi, Benin; 2 Institut de Recherche pour le Développement, UMR 216, Mère et enfant face aux infections tropicales, Paris, France; 3 PRES Sorbonne Paris Cité, Université Paris Descartes, Faculté de Pharmacie, France; 4 Department of Immunology, Wenner-Gren Institute, Stockholm University, Stockholm, Sweden; 5 Department of Hematology and Oncology, University of Bologna, Italy

## Background

Placental malaria (PM) is caused by sequestration of *Plasmodium falciparum* infected erythrocytes into the intervillous space of the placenta, resulting in pathological alterations. During PM, mononuclear cells infiltrate the placenta inducing several immunological events, which cause pathological alterations in the placenta, impairing materno–fetal interaction. Several studies have shown transient depression of cell-mediated immunity of the woman during pregnancy and their modulation during PM. In this study, we investigated the impact of *P. falciparum* infection during pregnancy on inflammatory immune responses.

## Methods

We conducted a longitudinal, prospective study in Benin, in which we enrolled ~1000 pregnant women with a gestational age of 24 weeks or less, and followed them up until delivery. Immunophenotype and activation levels *ex vivo* of peripheral blood mononuclear cells (PBMC), as well as plasma concentrations of a panel of cytokines and chemokines, were assessed in subgroups of 132 women at inclusion and 111 at delivery, using flow cytometry, standard cytometric bead arrays and ELISA. *P. falciparum*-infected women were matched to uninfected controls based on age, gestational age and gravidity.

## Results

Both at inclusion and at delivery *P. falciparum* infection was associated with significantly increased frequencies both of B cells overall and of activated (CD86^hi^) B cells. Infection-related profiles were otherwise quite distinct at the two different time-points, characterized by, for example, fewer T regulatory cells (Treg) at inclusion but more T effector (Teff) cells at delivery. Independent associations with an increased risk of maternal anaemia were found for altered antigen-presenting cell frequencies at inclusion, but for an increased frequency of Teff at delivery. *P. falciparum* infection was also associated with increased IL-6, IL-10, MIG and IP-10 plasma levels both at inclusion and at delivery.

## Conclusions

The timing and/or duration of *P. falciparum* infections during pregnancy – chronic at inclusion but acute/recently-acquired at delivery – is reflected by similar (e.g. B cells) but also distinct (e.g.Treg/Teff, immature moncocytes) variations in PBMC populations. The data suggest that innate immune responses as well pro- and anti-inflammatory mediators play important roles in pregnancy- associated malaria pathogenesis.

